# Population Genomics and Structure of the Critically Endangered Mariana Crow (*Corvus kubaryi*)

**DOI:** 10.3390/genes10030187

**Published:** 2019-03-01

**Authors:** Nandadevi Cortes-Rodriguez, Michael G. Campana, Lainie Berry, Sarah Faegre, Scott R. Derrickson, Renee Robinette Ha, Rebecca B. Dikow, Christian Rutz, Robert C. Fleischer

**Affiliations:** 1 Center for Conservation Genomics, Smithsonian Conservation Biology Institute, National Zoological Park, 3001 Connecticut Ave., NW, Washington, DC 20008, USA; CampanaM@si.edu (M.G.C.); derricksons@outlook.com (S.R.D.); dikowr@si.edu (R.B.D.); FleischerR@si.edu (R.C.F.); 2 Department of Biological Sciences, Ithaca College, 953 Danby Rd, Ithaca, NY 14850, USA; 3 Department of Land and Natural Resources, Honolulu, HI 96813, USA; lainie.berry@hawaii.gov; 4 Department of Psychology, University of Washington, Seattle, WA 98195, USA; sfaegre@gmail.com (S.F.); robinet@u.washington.edu (R.R.H.); 5 Data Science Lab, Office of the Chief Information Officer, Smithsonian Institution, Wahington, DC 20560, USA; 6 Centre for Biological Diversity, School of Biology, University of St Andrews, Sir Harold Mitchell Building, St Andrews KY16 9TH, UK; christian.rutz@st-andrews.ac.uk

**Keywords:** Mariana Crow, SNPs, Population genomics, Conservation, Inbreeding, Population decline

## Abstract

The Mariana Crow, or Åga (*Corvus kubaryi*), is a critically endangered species (IUCN -International Union for Conservation of Nature), endemic to the islands of Guam and Rota in the Mariana Archipelago. It is locally extinct on Guam, and numbers have declined dramatically on Rota to a historical low of less than 55 breeding pairs throughout the island in 2013. Because of its extirpation on Guam and population decline on Rota, it is of critical importance to assess the genetic variation among individuals to assist ongoing recovery efforts. We conducted a population genomics analysis comparing the Guam and Rota populations and studied the genetic structure of the Rota population. We used blood samples from five birds from Guam and 78 birds from Rota. We identified 145,552 candidate single nucleotide variants (SNVs) from a genome sequence of an individual from Rota and selected a subset of these to develop an oligonucleotide in-solution capture assay. The Guam and Rota populations were genetically differentiated from each other. Crow populations sampled broadly across their range on Rota showed significant genetic structuring – a surprising result given the small size of this island and the good flight capabilities of the species. Knowledge of its genetic structure will help improve management strategies to help with its recovery.

## 1. Introduction

The geographic isolation of oceanic islands makes them useful for studying basic ecological and evolutionary processes, such as population dynamics, adaptive radiation, speciation, and community assembly [1,2]. Island species are more vulnerable to extinction than their mainland counterparts, due to a combination of factors, including relatively small population sizes, low genetic diversity, and increased vulnerability to invasive species [3]. In fact, over 90% of all recorded bird extinctions in the world over the last four centuries have involved insular species [4]. As in most other island groups, the avifauna of the Mariana Islands, an archipelago of 15 volcanic-origin islands in the western Pacific Ocean [5], has been altered substantially since human colonization, as a result of the introduction of invasive species, overharvesting and habitat modification and degradation [6,7,8].

Within the Mariana Archipelago, forest bird populations have been declining from several causes, notably the introduction of the Australasian brown tree snake (*Boiga irregularis*) to Guam after World War II [9,10,11]. By 1969, this invasive species had spread throughout the island, but it was not until the 1980s that most of the bird populations had significantly declined or disappeared [12]. The brown tree snake has been responsible for extirpating or greatly reducing the native populations of the Guam rail (*Rallus owstoni*; [13]), the Mariana fruit bat (*Pteropus mariannus*; [5]), the Micronesian kingfisher (*Todiramphus cinnamominus*; [14]), the Mariana swiftlet (*Aerodramus bartschi*; [15]), and the Mariana Crow or Åga (*Corvus kubaryi*).

**Figure 1:**
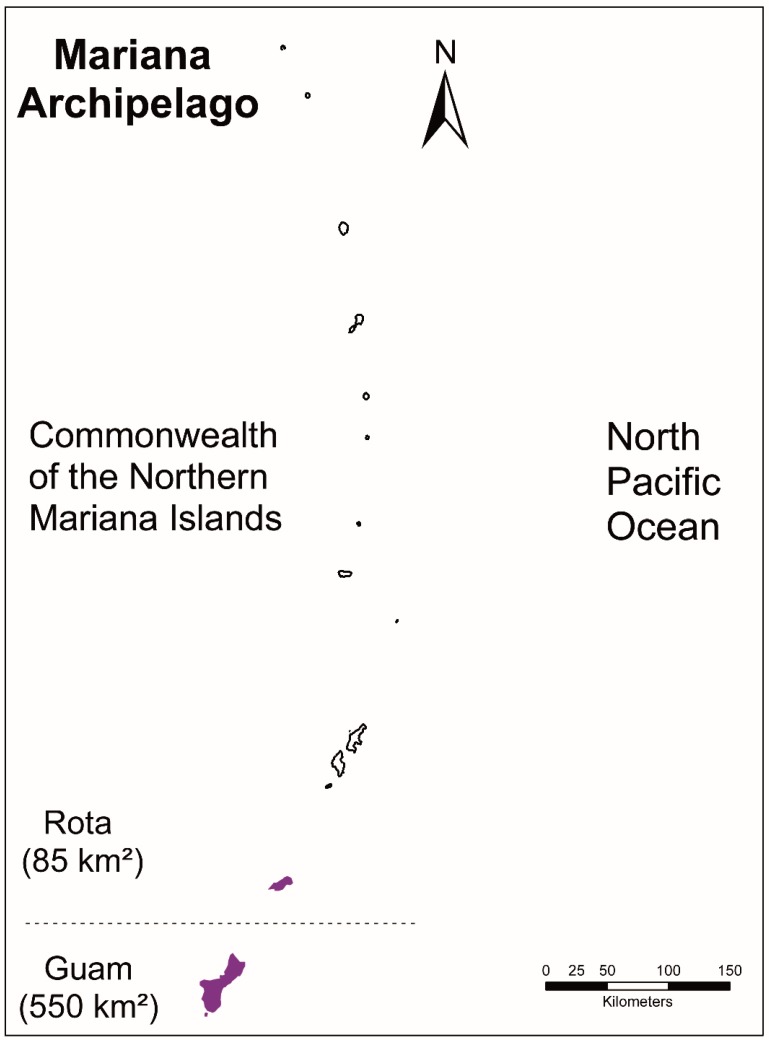
Map of the Mariana Archipelago and the historical distribution colored in purple of the Mariana Crow.

The Mariana Crow is a critically endangered species endemic to the two southernmost islands in the Mariana Archipelago: Guam (540 km^2^) and Rota (85.2 km^2^ ~49 km NE of Guam, Figure 1). By 2011, the Mariana Crow was extirpated from Guam despite avicultural intervention and supplementation with 25 birds (7 adults, 18 juveniles) from Rota between 1997 and 2005 [12,16,17]. The Mariana Crow presently survives only on Rota, where the population has declined 46–53% since 1988, and 10–23% between 2007 and 2014 (Table 1; [3,18,19,20]). The principal factors for this dramatic decline included: cat predation, disease, human disturbance and persecution, and both human and typhoon-related habitat modifications, all of which have likely contributed to its low genetic variability [21]. In addition, there is still concern that the brown tree snake could arrive on Rota via shipments from Guam and become established [12].

In a first study on Mariana Crow population genetics, Tarr & Fleischer [21] found that the Guam and Rota populations differed by two base pairs in mitochondrial DNA (mtDNA) control region sequences and slightly, but significantly, in microsatellite allele frequencies. Genetic variability in both populations was very low, suggesting that the species had undergone a genetic bottleneck. The variability of the Rota population was comparatively lower than that of the Guam population, indicating that it had experienced a more dramatic change, either through a reduction in population size to just a few individuals, or a recolonization event comprising a relatively small number of founders from Guam [21].

Furthermore, while the cause of low nest success on Rota (25.7%, n = 204 nests over 14 years) is currently unknown, low genetic variability and potentially high levels of inbreeding may be contributing factors [28,29,30]. To prevent the species’ extinction, a range of management strategies are currently being explored, including: (a) harvesting of eggs and/or chicks from the wild for captive rearing and subsequent release into the wild; (b) establishing a captive breeding flock for supplementing the wild population; and (c) translocating birds to another island with similar habitat. Evaluating kinship levels will inform selection of individuals for these programs. Also, it will be of particular interest to know if dispersal barriers are preventing Mariana Crows from reaching optimal habitat and unpaired partners.

In our study, we sequenced and assembled the genome of a single Mariana Crow from Rota and subsequently identified 145,552 putative single-nucleotide variants (SNVs), including 101,040 high-quality candidate polymorphisms. We selected a subset of 19,991 of these markers to use for the synthesis of an in-solution oligo capture bait set that could be used to assess variation in Mariana Crows. Here, we use these resources: (a) to compare genetic variability and assess differentiation between crows on Guam and Rota; (b) to chart the genetic structure of crow populations across different regions of Rota; (c) to assess possible patterns of dispersal from genetic structure data; and (d) to estimate preliminary levels of kinship among individual birds on Rota.

## 2. Materials and Methods 

### 2.1. Crow Samples 

We collected blood samples from wild Mariana Crows on Rota throughout their range from recent conservation research projects (2009–2015), performed by the University of Washington and funded by the Commonwealth of the Northern Mariana Islands Department of Land and Natural Resources (CNMI DLNR). Samples were obtained by venipuncture from nestlings, fledglings and subadults (n = 51), and adults (n = 13). In addition, we included 13 samples (5 from Guam, 8 from Rota) from the 1990s, which had previously been analyzed by Tarr & Fleischer [21,31]. An additional six samples came from Guam’s Division of Aquatic and Wildlife Resources and were also collected in the 1990s. Blood was mixed with lysis buffer using the Puregene DNA Purification Kit protocol and stored at −20 °C. While we obtained samples from 99 Mariana Crows, only a subset of them (83 individuals: 5 from Guam, 78 from Rota) had sufficient DNA or yielded enough sequence coverage to be included in further analyses (see Supplementary Materials Table S1).

### 2.2. Genome Sequencing and Assembly 

As part of a separate ongoing comparative genomics study of the genus *Corvus*, we sequenced the complete genome of an individual male Mariana Crow from Rota (Crow ID #213145, originally sampled on 11 July 1994 while held in captivity at the Smithsonian Conservation Biology Institute - SCBI) on an Illumina HiSeq (2 × 143 paired end sequences, ~500 bp library insert size). We trimmed the reads using Trimmomatic 0.33 ILLUMINACLIP:TruSeq3-PE.fa:2:30:10, LEADING: 3, TRAILING: 3, SLIDINGWINDOW:4:28, MINLEN:36). We used FastQC 0.11.2 to check read qualities and ensure that adapter sequences were removed [32]. Trimmed reads were then assembled with MaSuRCA [33], using their suggested parameters for eukaryotic genomes (cgwErrorRate:0.15, LIMIT_JUMP_COVERAGE = 300). Using BEDTools 2.2.23 genomecov and a custom script (available: https://github.com/campanam/genome_coverage), we estimated genome coverage as 33.4x [34]. Genome assembly contiguity statistics were calculated using a custom script (available: https://github.com/pbfrandsen/fasta_metadata_parser). We estimated genome completeness using BUSCO v3 [35] with the Aves_odb9 reference gene set. The following steps were performed to annotate the genome. We first used RepeatMasker [36] to detect and categorize repeats in the genome. We also aligned *Corvus cornix* transcripts (from Genbank assembly ASM73873v2) to the genome with BLAT and used the blat2hints Augustus utility to create a hints file for training Augustus. We then ran Augustus with RepeatMasker and BLAT hints (v3.3 [37]) twice, first using chicken (*Gallus gallus*) as the model species, and then using the BUSCO-generated crow-specific Augustus retraining gene models. A consensus GFF was generated with EVidenceModeler [38].

### 2.3. Hybridization Capture Assay Design 

The sequenced individual’s trimmed reads were re-mapped against the assembled genome using BWA-MEM 0.7.12 [39]. We removed duplicate reads using Picard 1.138 MarkDuplicates [40]. Sequence variants were identified using SAMtools 1.2 mpileup (-C50) and BCFtools 1.2 call (-m) [41], yielding 145,552 candidate single nucleotide variant (SNVs). We identified 101,040 high-quality SNVs using BCFtools filter with the following requirements: 15 < DP < 110, QUAL >= 30, 0.3 < AF < 0.7. We selected 19,991 SNVs and generated 120 bp bait sequences to capture these markers using select snps 0.3 (now BaitsTools [42]) under default settings (no more than 2 SNVs per contig with a minimum distance of 10,000 bp separating them). The sequences were synthesized as a custom MYBaits-1 capture kit (Arbor Biosciences, formerly known as MYcroarray, Inc.). Sequences are available in the Supplementary Materials Data.

### 2.4. DNA Extraction, Library Preparation, Capture and Next Generation Sequencing 

We extracted DNA from the blood samples using Qiagen DNeasy Blood & Tissue kits (Qiagen, Valencia, CA, USA). DNA samples were sheared to ~500 bp using a Q800R sonicator (Qsonica, LLC, Newtown, CT, USA) and converted into double-indexed Illumina libraries using KAPA Library Preparation kits – Illumina (Kapa Biosystems, Wilmington, MA, USA). Library concentrations were quantified using Qubit^®^ 2.0 dsDNA HS assays (Thermo Fisher Scientific, Waltham, MA, USA) and library size-ranges and qualities were inspected using 2100 Bioanalyzer (Agilent Technologies, Santa Clara, CA, USA) high-sensitivity DNA chips. Libraries were pooled at the same concentration in groups of four to six individuals and hybridized to the MYBaits capture kit (1.25 µL baits per capture) for ~24 h according to the MYBaits protocol v. 3.0. Finally, pooled libraries were 2 × 150 bp paired-end sequenced on a HiSeq 2500 (Illumina, Inc., San Diego, CA, USA) at the Tufts University Core Facility (TUCF) genomics services (Tufts University School of Medicine, Boston, MA) and using a MiSeq (Illumina, Inc., San Diego, CA, USA) at the Center for Conservation Genomics (National Zoological Park, Washington, DC). Supplementary 2 × 123 bp paired-end Illumina HiSeq sequencing was performed at RTL Genomics (Research and Technology Laboratories, Lubbock, TX).

### 2.5. Sequence Alignment and Genotyping

Samples were demultiplexed using the BaseSpace (Illumina, Inc.) pipeline or deML (option --m 1) [43]. Library qualities were checked using FastQC 0.11.4 [32]. We trimmed the reads using Trimmomatic 0.36 (ILLUMINACLIP:Nextera-PE.fa:2:30:10, LEADING: 3, TRAILING: 3, SLIDINGWINDOW:4:20, MINLEN:36) [44], and merged the resulting reads using FLASH 1.2.11 (option -M151 or -M123 depending on maximum read length; [45]). The merged and unmerged reads were aligned against the Mariana Crow genome, using BWA-MEM 0.7.12 [39] and deduplicated using SAMtools 1.3 [41]. Read groups were added using Picard 2.9.4 AddOrReplaceReadGroups [40]. Indels were left-aligned using the Genome Analysis Toolkit 4.0.7.0 LeftAlignIndels [46].

Sequence variants within the baited regions were identified using BCFtools 1.9 mpileup (options -q 30 -d 250 -t DP,AD) and BCFtools call (options -m -v). We removed non-SNVs and SNVs below quality 20 using BCFtools filter to obtain a dataset of 37,272 SNVs (some baited regions contained multiple SNV sites). Using VCFtools 0.1.15 [47], we further filtered the 37,272 SNVs to include only biallelic sites with a minimum mean depth per individual per site of 10 and no more than 5% missing data. Sites (n = 817) that deviated from Hardy-Weinberg equilibrium (α = 0.01) were excluded to reduce the impact of genotyping errors such as misalignments due to gene duplications. We limited linkage disequilibrium by choosing only one SNV per 120 bp bait. The final dataset included 6741 high-quality SNVs. The final SNV dataset was converted from VCF to Structure and PLINK formats using PGDSpider 2.1.1.2 [48] and PLINK 1.9033 [49], respectively.

### 2.6. Population Structure

We performed population structure analyses on two datasets: (a) the dataset including all 83 individuals from both Guam and Rota (“complete dataset”); and (b) a restricted dataset including only the Rota birds (“Rota-only dataset”) of known provenance (n = 72), to help resolve population substructure on Rota. Using SNPRelate 1.14.0 [50] in R 3.5.0 [51], we performed principal component analysis (PCA) on the two datasets and constructed dendrograms depicting the genetic similarity (Identity-by-State [IBS]) of the Mariana Crow individuals. To investigate the possibility of a genetic cline on Rota, we calculated genetic distances (1 - IBS) using SNPRelate and used this distance matrix to perform an isolation-by-distance analysis on the Rota-only dataset using the R package ADE4 1.7.11 (Analysis of Ecological Data: Exploratory and Euclidean Methods in Environmental Sciences [52]).

We inferred population structure in the complete dataset using the admixture model in Structure 2.3.4 [53] under default settings with 50,000 burn-in and 100,000 data collection steps. We assumed *K* values between 1 and 10, with three replicate runs per *K* value. We identified optimal *K* values using the **Δ***K* [54] and **Δ***F*_ST_ (both using no optimization and optimization by *Q*-matrix correlation) statistics, as well as *Q*-matrix correlations (both row-and-column and average maximum correlation methods) in CorrSieve 1.6-6 [55]. We repeated the Structure analysis using the Rota-only dataset (*K* = 1 to 10, four replicates per *K*) to infer fine-scale population structure on this island. Since fine-scale population structure can be more difficult to resolve using Bayesian approaches, we increased the run lengths to 100,000 burn-in and 200,000 data collection steps for this analysis. As an alternative methodology to infer population structure, using Adegenet 2.1.1 [56], we performed Discriminant Analysis of Principal Components (DAPC) on the two datasets. We evaluated the results of the DAPCs using Bayesian Information Criterion (BIC) analysis of the *K*-means clustering, retaining all components for each analysis. To avoid overfitting, we determined the number of retained principal components using the optimized *a*-score and cross-validation as implemented in Adegenet [57]. Cross-validation was performed using 70% of the data as the training data and the remaining 30% as the test data, 30 replicate runs, the groups assigned by initial *K*-means clustering at *K* = 3 (complete dataset) or *K* = 2 (Rota-only dataset), and two discriminant functions. We also assayed population structure in the two datasets using ADMIXTURE 1.3.0 for *K* = 1 to 10 under default settings except that the random number seed was set to the system clock and we used ten-fold cross-validation to identify the best *K* values [32].

### 2.7. Kinship and Inbreeding Analysis

We calculated mean kinship coefficients between individuals using maximum likelihood estimation in SNPRelate 1.14.0 [50] and custom code (available: https://github.com/campanam/kinshipUtils). We calculated the 95% confidence intervals for the kinship estimates using 100 bootstrap replicates. The kinship analysis included 5872 SNVs with a minor allele frequency greater than 0.05. We identified all individuals roughly on second order of kinship (*r* > 0.1) to be compared against individuals of known relatedness. Inbreeding coefficients (*F*_IS_) were calculated using VCFtools 0.1.15 [47].

## 3. Results

### 3.1. Genome

The assembled genome was 1.07 Gb long, similar to previously assembled passeriform genomes. The assembly N50 was 79,541 with a longest contig of 677,288 bp and a mean contig length of 19,865 bp (Table 2). The genome’s GC content was 42.22%. Genic content was complete with 89.5% (4396) of the 4915 Avian BUSCO orthologs being recovered completely (of which 1.0% (47) were duplicated). An additional 6.8% (334) of orthologs were fragmented, and 3.7% (185) were missing. Further analysis of the Mariana Crow genome (e.g., phylogenomic placement within the genus *Corvus*, further assessments of genome assembly quality, and functional genomics) will be presented in upcoming publications (consortium, including C. Rutz and R. C. Fleischer).

### 3.2. Single Nucleotide Variants Data

We generated analyzable sequencing data for 83 individual Mariana Crows (Guam n = 5; Rota n = 78). We sequenced between 130,968 and 10,400,122 read pairs per analyzed individual (mean of 2,299,326 read pairs) (Supplementary Materials Table S1). Raw data have been deposited in the NCBI Sequence Read Archive (accession SRP139974) in BioProject PRNJA448444 (NCBI BioSamples SAMN08910188- SAMN08910271). We excluded the remaining 16 individuals from our analyses due to insufficient sequence data. While we found that the patterns observed in our data were robust even under lenient filtration parameters (data not shown), we stringently filtered our variant calls to ensure high-quality SNV data despite variation in sequencing effort and sample quality. The final data occupancy matrix was very complete (mean and median per-sample genotype missing rates of 1.57% and 0.01%, respectively, with a standard deviation of 4.25%) (Supplementary Materials Table S1). One Guam specimen (Guam-55#1) had a high per-sample genotype missing rate (25.60%) in our analyses. However, since only five samples were available from the now-extinct Guam population, we included all Guam individuals in the analyses.

### 3.3. Principal Component Analysis

**Figure 2:**
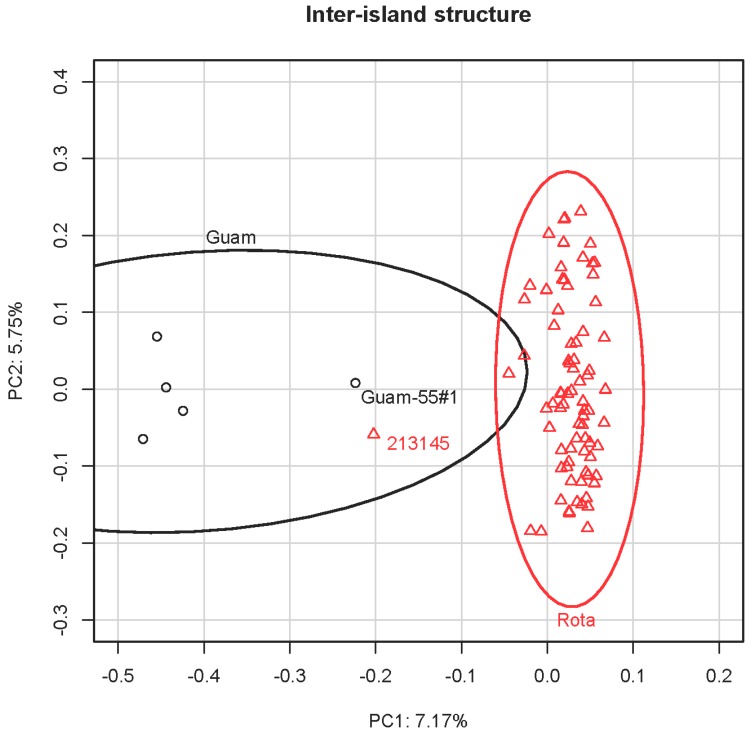
Principal Component Analysis (PCA) of Mariana Crows from Guam and Rota using 6741 SNVs. The 95% data ellipses clearly distinguish the populations from the two islands. Potentially (but see text for alternative interpretation) admixed individuals (Guam-55#1 and 213145) are denoted on the plot.

The first axis (encompassing 7.17% of the variation) of the PCA including the 83 crow samples differentiated the Rota and Guam populations from each other (Figure 2). There were two individuals (213145 from Rota and Guam-55#1 from Guam) roughly mid-way between the two island-specific clusters, suggestive of mixed ancestry. However, Rota individual 213145 was the individual from which the original baits were designed and therefore is far more heterozygous (HO = 0.873) than other individuals (Mean Rota HO = 0.259; Mean Guam HO = 0.333) due to ascertainment bias. Moreover, Guam-55#1’s central position may be due to that individual’s high amount of missing data noted above. The PCA including only the Rota birds of known provenance (Supplementary Materials Figure S1) revealed slight evidence of population structuring on the first principal component (6.86% of the variation), and the second component (5.15% of the variation) separated the putatively mixed-heritage 213145 from the remaining birds. However, the modest sample sizes prevented confident separation of the Rota subpopulations via PCA, since the 95% data ellipses largely overlapped (Supplementary Materials Figure S1). 

### 3.4. Population Structure

**Figure 3:**
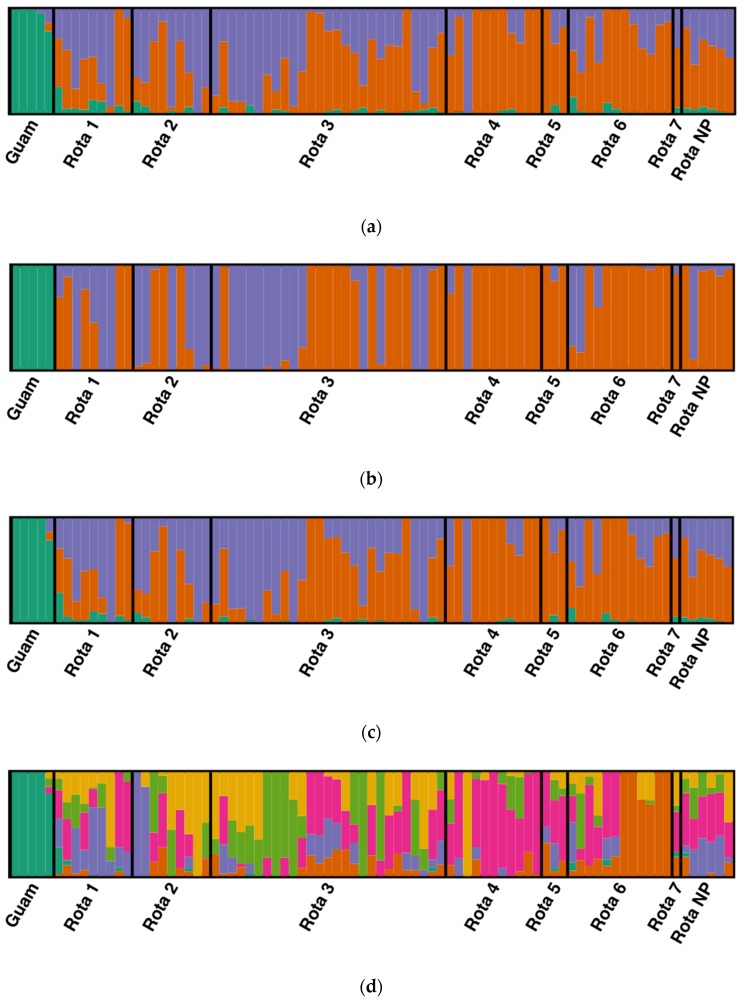
(**a**) Structure analysis showing the mapped Mariana Crow subpopulations of Rota (defined populations 1–7; see Figure 4) and Guam using *K* = 3. Population “Rota NP” includes Rota individuals of unknown provenance on the island. Each individual is represented by a vertical line partitioned into colored segments representing their proportional assignment to each of the three clusters. Orange and purple correspond to individuals from Rota, while green corresponds to individuals from Guam. (**b**) The Discriminant Analysis of Principal Components (DAPC) shows a very similar pattern to the Structure analysis. Individuals from Guam are differentiated (shown in green). In addition, most of the individuals from Northeast Rota form one group, while the southern portion of the island shows more mixing. (**c**) ADMIXTURE and Structure return nearly identical population assignments for *K* = 3. (**d**) ADMIXTURE identified some possible additional population structure under the *K* = 6 model. Figure drawn using Distruct 1.1 [58].

The optimal Structure solution for the complete dataset including all Mariana Crows was found at *K* = 3 populations by all methods in CorrSieve (Figure 3a). Occasionally, higher *K* values were supported by individual statistics, but these were not replicable. One population (green) corresponded to the five individuals from Guam, and the other two (orange and purple) only occurred on Rota. For the DAPC, the BIC analysis of *K*-means clustering also determined an optimal *K* of 3. The final DAPC retained 4 principal components (comprising 22.0% of the variation) and two discriminant functions. The DAPC cluster assignments largely agreed with those of the Structure analysis but showed less evidence of mixed ancestry within individuals (Figure 3b). While the ADMIXTURE population assignments for the complete dataset for *K* = 3 were nearly identical to those generated by Structure, the cross-validation (CV) error suggested that *K* = 6 (CV = 0.53560) was a slightly better solution than *K* = 3 (CV = 0.54818) for the ADMIXTURE analysis. The *K* = 6 model suggests some additional population substructure within Rota (Figure 3c,d). However, this apparent substructure is likely alternative partitioning of a genetic cline due to strong isolation-by-distance ([57]; see below). The Structure and ADMIXTURE analyses indicated that approximately 21% of the putatively admixed Guam-55#1’s ancestry derived from Rota populations.

The analysis of the Rota-only dataset did not detect further statistically reliable substructure. Both **Δ***K* and **Δ***F*_ST_ statistics indicated that the best Structure solution was at *K* = 2, although *Q*-matrix correlations found that some higher *K* values were reproducible. The optimal DAPC solution was also found at *K* = 2 by BIC analysis. The final Rota-only DAPC retained four principal components (comprising 22.2% of the variation) and one discriminant function. The optimal ADMIXTURE solution was found at *K* = 5 (CV = 0.51973). These results are consistent with those of the complete dataset with the Guam population removed.

**Figure 4:**
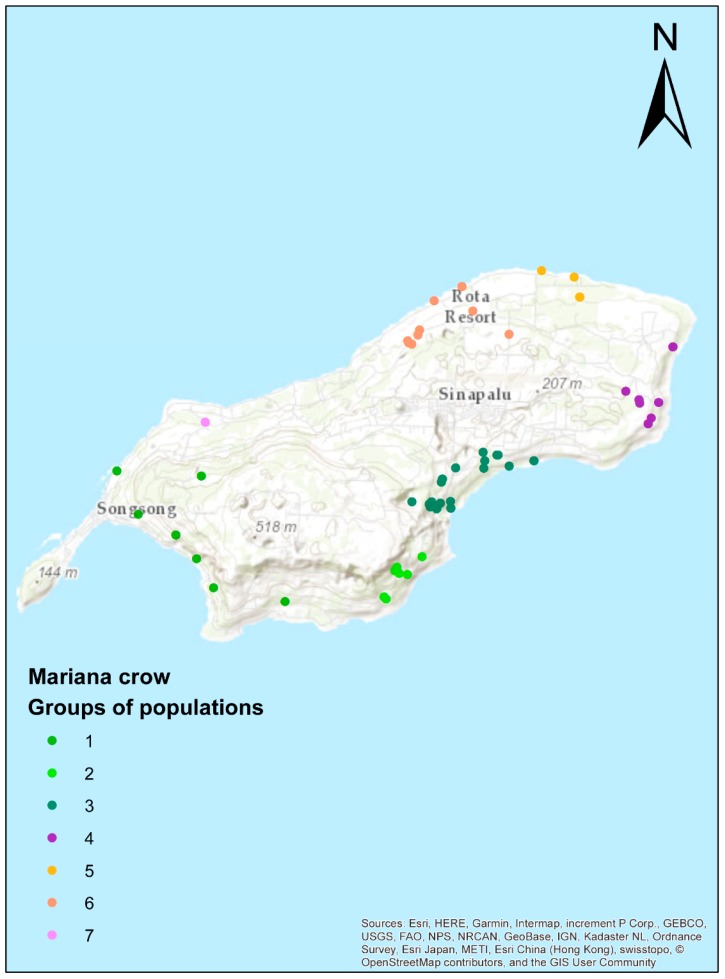
Assignment of Rota crows to populations based on their known provenances. Each dot represents a single individual and is colored by its population assignment.

**Figure 5:**
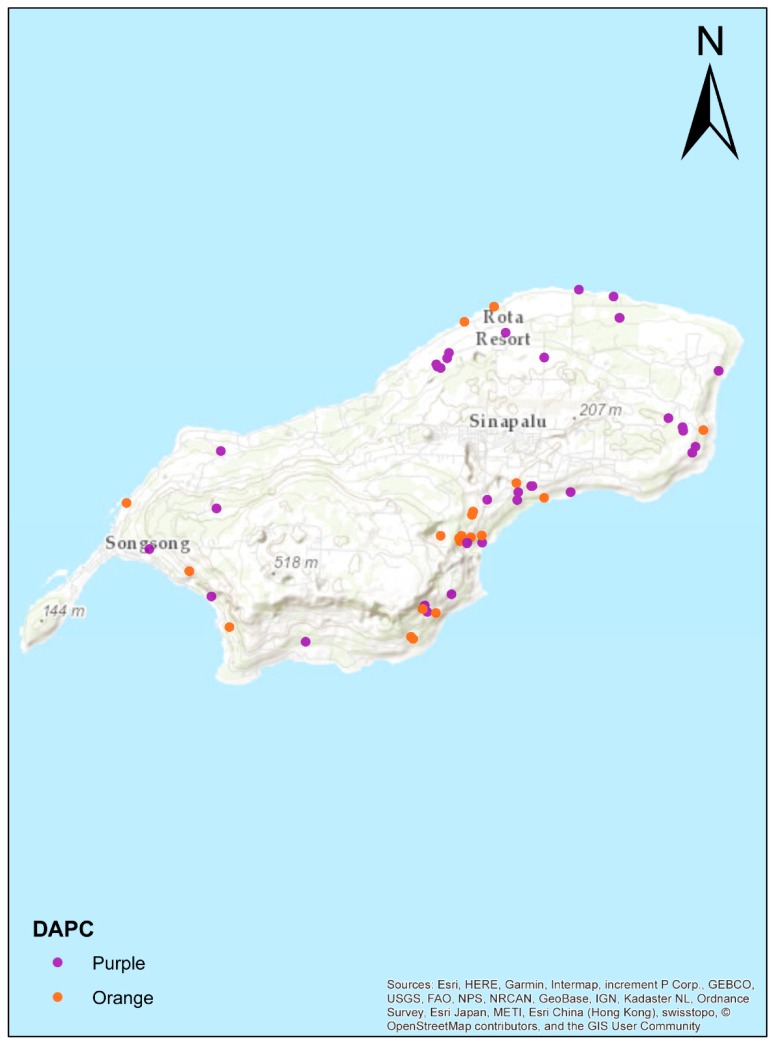
Map of the Rota Mariana Crow individuals obtained with DAPC (Figure 3b, note that Guam individuals are in green – not shown in map). Note that the North and East coasts are nearly all designated by DAPC to fall in the purple cluster, while the South and West coasts are more evenly mixed. This suggests isolation of the northeastern crow populations, with some mixing of these birds into the southwestern populations.

To gain a better understanding of the Rota crows’ known genetic relationships, we plotted each sample on a map of Rota, and grouped them by proximity based on their geographic location (Figure 4, Supplementary Materials Table S1). The two Rota clusters from the structure analysis showed a geographic pattern suggesting a genetic gradient running from Southwest to Northeast (Figure 5). Southern and western populations (1–3) were more admixed than northern and eastern populations (4–7) (unpaired t-tests, Structure and ADMIXTURE: *p* = 0.0001, DAPC: *p* = 0.0002). The dendrogram clustered the individuals from Guam and Rota into two separate clades (Supplementary Materials Figure S2); however, it does not provide strong evidence of population and family structure within the Rota sample. The isolation-by-distance analysis also supported a genetic gradient by showing a highly significant correlation between geographic and log-transformed genetic distances (number of permutations = 9999, *r* = 0.296, *p* < 0.001). 

### 3.5. Kinship and Inbreeding Analysis

We found relatively low estimated values of kinship (Supplementary Materials Table S2) for most pairwise comparisons. The reference Rota individual 213145 showed roughly second-order kinship (~0.1) with all Guam birds, except Guam-55#1, reflecting ascertainment bias or possible admixture. Conversely, despite its central location within the PCA, Guam-55#1 did not have close kinship with any Rota birds, suggesting its central location may be due to high sample missingness. Nevertheless, admixed ancestry for Guam-55#1 cannot be ruled out. Individuals 99404745 and 84477245 had an extremely high kinship value (0.453), indicating that they may be the same individual or potential siblings. However, the two birds were banded two years apart at different locations: 84477245 was radio- tagged as a fledgling in 2012 at Finata and found dead 25 days later, while 99404745 was banded as a nestling in 2014 at Agatasi and was last seen in November 2017. Since there is a possibility of a sample mix-up, we re-ran all analyses from genotyping onwards excluding these two individuals. The results were nearly identical except that ADMIXTURE found that *K* = 5 was the best solution for the complete dataset (Supplementary Materials Table S1 and S2, Supplementary Materials Figures S1, S3 and S4).

The inbreeding coefficient (*F*_IS_) was significantly higher (unpaired *t*-test, *p* = 0.0272) for individuals from Guam (mean *F*_IS_ = 0.254, standard deviation = 0.0577) than for those on Rota (mean *F*_IS_ = 0.024, standard deviation = 0.227). However, the Rota birds exhibited substantial variation in their inbreeding coefficients, with some birds being highly inbred (24 individuals with *F*_IS_ > 0.1) and others being very outbred (13 with *F*_IS_ < −0.1).

## 4. Discussion

Efforts to conserve island bird populations have a long tradition. Although extinction rates remain high, there have been a number of notable success stories, thanks to active conservation measures, including the eradication of alien species, habitat restoration, captive propagation, and the relocation of populations [2]. In the first study on Mariana Crow genetics, conducted some 20 years ago, birds from Rota showed significantly lower genetic diversity in microsatellite profiles and mtDNA haplotypes compared to individuals from nearby Guam [31]. In contrast, our present SNV results indicate higher genetic variability in Rota samples than Guam samples, which could indicate: (a) a more severe contemporary genetic bottleneck on Guam (e.g., as a result of snake- related declines); (b) a greater disparity in sample sizes (8 and 8 in [31], versus 5 and 78 in the present study); or (c) differences in the statistical power afforded by the different molecular markers chosen for detecting bottlenecks [59]. In addition, we found a clear genetic differentiation between the Rota and Guam populations, which was expected based on earlier results [31], and probably reflects limited movement of individuals between islands.

Despite the observed genetic variation among Rota individuals, a future genetic bottleneck remains possible, given the few (55) numbers of breeding pairs remaining in the wild (75 is the estimated number of breeding pairs needed to maintain a viable population [16,18]). Furthermore, the observed structure on Rota might be due to higher crow densities on the southern side of the island, and limited movement between northern and southern regions [18]. The median distance between natal and breeding nests for 42 banded crows on Rota was only 1.6 km (range = 96 m to 12,687 m), with females (n = 25) and males (n = 17) dispersing a median of 1.6 km and 1.3 km, respectively [60,61]. Moreover, home range estimates on Rota, using Minimum Convex Polygons (MCP), are an average of 76 hectares for family groups, and 136 hectares for sub-adults [62]. This is much smaller than the ranges reported for some mainland corvids, such as American crows (*Corvus brachyrhynchos* - MCP; adults, 640 hectares; sub-adults, 670 hectares; [63]) or common ravens (*Corvus corax* - sub-adult males, 14200 hectares; sub-adults females; 18800 hectares; [64]). The New Caledonian Crow (*Corvus moneduloides*), an island endemic from New Caledonia in the South Pacific, also maintains small home ranges [65]. Smaller home ranges in Mariana Crows may result from a number of factors: (a) the Mariana Crow inhabits a much smaller landmass and therefore has smaller territories compared to continental birds; (b) differences may be driven by ecological conditions; or (c) Mariana Crows are smaller than American crows or Common ravens, which could imply a smaller territory [66].

Our isolation-by-distance analysis suggests that most of the pattern of the genetic differentiation is due to the distance between populations from the northern and southern portions of the island. There is only one documented dispersal between the north and south sides of Rota: a female dispersed 12,687 m while all other dispersals were less than 5000 m [67]. While corvids are generally thought to be capable of long-distance movements, in a number of species a surprising degree of small-scale population structure has been detected [68,69,70,71]. One species that has a high dispersal capacity, yet unusual genetic structure, is the Red-billed Chough (*Pyrrhocorax pyrrhocorax*) in the Iberian Peninsula. Morinha and collaborators [72] found that, even though the Red-billed Chough is capable of travelling for hundreds of kilometers, their breeding behavior is highly philopatric. In the New Caledonian Crow, some populations show significant genetic differentiation over distances <10 km [70], associated with surprisingly small movement ranges [72] and notable variation in behavior [73].

Several factors continue to impact the Mariana Crow on Rota, such as habitat loss due to land use conflicts, disease and feral cat predation [16,17,61]. Thus, continued efforts to address these issues and improve the public perception of these crows should be major conservation focuses [17,18]. In addition, Ha and collaborators [74] compared first-year mortality across a range of corvids, and found that Mariana Crows showed a decline of survival rate from 70% to 40% over a ten year period, which is lower than needed to maintain a stable population, and even below that observed in the ‘Alalā or Hawaiian Crow (*Corvus hawaiiensis* - 43% [73]) – another tropical island corvid whose wild populations were supplemented with juveniles raised from wild-origin and captive-origin eggs in the early 1990s [28]. While this effort to sustain the remnant ‘Alalā population failed, more recent reintroductions in 2017 and 2018, which incorporated pre-release anti-predator training, have achieved excellent rates of survival.

Our genomic analyses imply that even though this species is critically endangered and has low genetic variability the extant Rota population does not appear to be highly inbred. Nonetheless, it is still important to assess the potential role of inbreeding in the low nesting success of Mariana Crows on Rota, especially since inbreeding coefficients show substantial variation among individuals. Some wild pairs have not produced viable young from their eggs, and some eggs harvested from wild pairs have proved to be inviable. This crow population not only exhibits high first-year mortality [74], but also low egg viability and infertility, which may contribute to its decline [16]. Furthermore, the Guam population became extremely inbred immediately before its extinction [16], which is a possibility in the Rota population without careful management.

Similar to the Mariana crow, the ‘Alalā or Hawaiian Crow, experienced a population crash in the last century, eventually becoming extinct in the wild in 2002 [75]. Fortunately, extinction was averted, as some birds had been brought into captivity over the years, to breed stock for reintroductions [75]. While the captive flock has gradually grown to well over 100 individuals, it descended from a mere nine genetic founders. To inform the long-term genetic management of the species, and ongoing releases, researchers have recently conducted pedigree-based analyses to examine patterns of founder representation and inbreeding depression [28,29], and sequenced a reference genome for forthcoming analyses [30]. For the Mariana Crow, we have presented here a detailed assessment of population structure, inbreeding and kinship levels, using cutting-edge genomics techniques that will enable effective management of dwindling extant populations. One management technique can be mixing the sub-populations through captive rear- and-release.

In a recent effort to increase the population, nine Mariana Crows were collected as eggs or chicks, early in the breeding season, allowing wild pairs to re-nest during the same season. Eggs and nestlings were collected from multiple sub-populations and reared in captivity for two years to protect them during the period of high mortality in wild birds. These individuals were released in September 2018 as reproductively mature adults on the north end of the island (Lalayak) where population declines have left large areas of vacant crow habitat. Eight of these birds were collected from the wild as eggs or nestlings, and one was taken into rehabilitation as a fledgling. Five of these birds came from southern areas of the island, so this effort will potentially increase genetic diversity within the Lalayak subpopulation.

## 5. Conclusions

The Mariana Crow is a prime example of a species that is critically endangered because of invasive species, diseases, human persecution and habitat degradation. It was extirpated from Guam in 2011 and the Rota population has declined dramatically over the past 31 years. Genetic analyses are an essential prerequisite for implementing effective management strategies for severely bottlenecked bird populations. Using high-quality SNV data, we found that, surprisingly, the Rota population does not yet exhibit high levels of inbreeding, suggesting that it is not too late to preserve extant genetic diversity. In wild populations, high first-year mortality, low egg viability, and infertility of some adults are major concerns. Nevertheless, despite the threats the species faces, with adequate management, it is still possible to prevent its extinction. The information we have presented on the genetic structure of the Rota subpopulations will enable managers to evaluate future release locations on the basis of genetic structuring.

## Figures and Tables

**Table 1:** Summary of results of Mariana Crow surveys on Rota since 1982. [Modified from Table 1 in the Revised Recovery Plan for the Mariana Crow [16]]. Variable circular plot is abbreviated as VCP.

Year	Est. Number (and/or Range of Individuals	Survey Method	Source
1982	1318 (1136–1564)	Off-road VCP	[20]
1988	(600–1000)	Informal Estimate	[22]
1992	(447–931)	Roadside VCP	[23]
1993	(336–454)	Roadside VCP	[23]
1995	592 (474–729)	Off-road VCP	[24]
1995	(365–607)	Off-road VCP	[25]
1998	(138–504)	Off-road VCP	[25]
1999	234 breeding adults	Extrapolated from known pairs and density estimates	[19]
2004	170 breeding adults	Extrapolated from known pairs and density estimates	[26]
2007	120 breeding adults	Direct count of breeding pairs	[3]
2012	(30–202)	Off-road VCP	[27]
2014	108 breeding adults; 178	Direct Count of pairs; Chapman estimate of total population	[18]

**Table 2:** Genome assembly statistics.

Statistic	Statistic	Statistic
Total base pairs: 1,073,754,616	N10: 229,841	L10: 363
Total number contigs: 54,052	N20: 168,264	L20: 915
Mean contig length: 19,865	N30: 128,954	L30: 1651
Median contig length: 2383	N40: 100,208	L40: 2597
Longest contig: 677,288	N50: 79,541	L50: 3800
Shortest contig: 182		

## Supplementary Materials

The following are available online at https://www.mdpi.com/2073-4425/10/3/187/s1, Figure S1: Principle Component Analysis of Rota subpopulations; Figure S2. Dendrogram using 6741 SNVs showing a clear separation between Mariana Crow samples from Guam (n = 5, black dots) and Rota (n = 78, red dots); Figure S3. Principal Component Analysis (PCA) of Mariana Crows from Guam and Rota using 6722 SNVs; Figure S4. Structure analysis showing the mapped Mariana Crow subpopulations of Rota (defined populations 1–7) and Guam using *K* = 3; Table S1: Location where each one of the Mariana Crows sequenced for this study; Table S2. Kinship estimates of all individual pairs; Supplementary Materials Data: Bait sequences to capture Mariana Crow polymorphic single nucleotide variants.
