# Supplementary material for: Population Genomics and Structure of the Critically Endangered Mariana Crow (Corvus kubaryi)
Source: Genes (Basel). 2019 Mar 1;10(3):187. doi: 10.3390/genes10030187 (PMC6471520; doi:10.3390/genes10030187)
Supplement: Supplementary file 1 [file genes-10-00187-s001.zip › SI_figures.pdf]

SI Table 1. Location where each one of the Mariana crows sequenced for this study. Values per read per individual are included as well as observed homozygosity, expected homozygosity and F_{IS} values. Tab “SampleInformation” gives statistics calculated using all individuals. “Statistics_84477245_99404745” lists statistics calculated with individuals 84477245 and 99404745 excluded.

SI Table 2. Kinship estimates of all individual pairs. Rota individual 213145 shows notable kinship with the Guam birds. Tabs “MeanKinships_AllIndividuals” and “MeanKinships_84477245_94404745” depict heat maps of mean kinship estimates with high kinship values in red and low values in white, while “KinshipCI_AllIndividuals” and “KinshipCI_84477245_99404745” give their corresponding 95% confidence intervals. “MeanKinships_AllIndividuals” and “KinshipCI_AllIndividuals” includes all individuals, while “MeanKinships_84477245_94404745” and “KinshipCI_84477245_99404745” excludes individuals 84477245 and 99404745.

SI Figure 1. Principle Component Analysis of Rota subpopulations. The top row uses all samples (6,741 SNVs), while the bottom excludes individuals 84477245 and 99404745 (6,722 SNVs) due to a possible sample mix-up. Although there is weak evidence of structuring demonstrated by the 50% data ellipses, the 95% data ellipses are largely overlapping, indicating that larger sample sizes are needed to confidently resolve populations via PCA.

SI Figure 2. Dendrogram using 6,741 SNVs showing a clear separation between Mariana Crow samples from Guam (n = 5, black dots) and Rota (n = 78, red dots). Genetic distances are based on identity-by-state.

SI Figure 3. Principal Component Analysis (PCA) of Mariana Crows from Guam and Rota using 6,722 SNVs. Individuals 84477245 and 99404745 have been excluded due to a possible sample mix-up. The 95% data ellipses clearly distinguish the populations from the two islands**.** The two intermediate individuals along Eigenvector 1 could be individuals of mixed Rota/Guam ancestry, with one on Guam and the other on Rota.

SI Figure 4. (a) Structure analysis showing the mapped Mariana Crow subpopulations of Rota (defined populations 1–7) and Guam using $K = 3$. Individuals 84477245 and 99404745 have been excluded due to a possible sample mix-up. Population “Rota NP” includes Rota individuals of unknown provenance on the island. Each individual is represented by a vertical line partitioned into colored segments representing their proportional assignment to each of the three clusters. Orange and purple correspond to individuals from Rota, while green correspond to individuals from Guam. (b) The Discriminant Analysis of Principal Components (DAPC) shows a very similar pattern to the structure analysis. Individuals from Guam are differentiated (shown in green). In addition, most of the individuals from Northeast Rota form one group, while the southern portion of the island shows more mixing. (c) ADMIXTURE and Structure return nearly identical population assignments for $K = 3$. ADMIXTURE identified some possible additional population structure under the $K = 5$ model. Figure drawn using DISTRUCT 1.1 (Rosenberg 2004).

SI Data. Mariana Crow (*Corvus kubaryi*) capture probe sequences in FASTA format.

Individual	Mean Site Depth	Missing Genotypes (% Missing)	Observed Homozygosity	Expected Homozygosity	F_{IS}
279	15.80	10 (0.14%)	4985	4398.4	0.258
281	11.64	12 (0.17%)	4988	4397.5	0.260
668	8.12	105 (1.56%)	4771	4334.6	0.194
901	111.89	2 (0.02%)	5131	4404.0	0.319
Guam-55#1	3.84	1729 (25.72%)	3578	3283.7	0.174
213142	25.45	1 (0.01%)	4543	4405.9	0.060
213143	23.21	0 (0.00%)	4153	4405.4	-0.111
213144	36.47	1 (0.01%)	4363	4405.0	-0.018
213145	16.48	3 (0.04%)	853	4403.5	-1.559
213146	47.87	0 (0.00%)	4394	4405.9	-0.005
213392	14.28	33 (0.49%)	4510	4382.8	0.056
213396	33.30	1 (0.01%)	4392	4405.2	-0.006
215119	3.87	826 (12.28%)	4306	3875.7	0.216
84477217	19.08	1 (0.01%)	4186	4405.1	-0.096
84477218	62.36	0 (0.00%)	4580	4405.9	0.076
84477219	25.29	0 (0.00%)	4508	4405.9	0.045
84477221	38.16	0 (0.00%)	4431	4405.9	0.011
84477222	12.22	12 (0.17%)	4455	4397.9	0.025
84477223	17.57	0 (0.00%)	4327	4405.9	-0.035
84477227	26.96	0 (0.00%)	4412	4405.9	0.003
84477230	84.37	0 (0.00%)	4159	4405.9	-0.108
84477231	53.83	0 (0.00%)	4397	4405.9	-0.004
84477232	48.77	0 (0.00%)	4838	4405.9	0.190
84477233	14.40	9 (0.13%)	4568	4399.0	0.074
84477234	22.68	0 (0.00%)	4138	4405.9	-0.118
84477235	19.00	3 (0.04%)	4238	4403.5	-0.073
84477236	39.76	0 (0.00%)	4590	4405.9	0.081
84477238	16.65	1 (0.01%)	4500	4405.3	0.042
84477239	76.97	0 (0.00%)	4538	4405.9	0.058
84477240	11.98	9 (0.13%)	4457	4400.2	0.025
84477243	22.82	1 (0.01%)	4601	4405.3	0.086
84477246	27.18	0 (0.00%)	4440	4405.9	0.015
84477247	45.39	0 (0.00%)	4425	4405.9	0.008
84477251	7.67	132 (1.96%)	5013	4315.7	0.312
84477254	42.33	0 (0.00%)	4381	4405.9	-0.011
84477255	16.42	1 (0.01%)	5181	4405.1	0.341
99403003	27.10	0 (0.00%)	4503	4405.9	0.043
99403004	24.74	0 (0.00%)	4494	4405.9	0.039
99403005	13.84	5 (0.07%)	4027	4402.3	-0.165
99403006	22.72	0 (0.00%)	4806	4405.9	0.176
99403007	18.05	0 (0.00%)	4837	4405.9	0.189
99403008	13.48	28 (0.41%)	3916	4387.4	-0.208
99403009	46.90	0 (0.00%)	4100	4405.9	-0.134
99403010	2.54	1140 (16.95%)	4098	3656.5	0.233
99403012	79.35	0 (0.00%)	4240	4405.9	-0.073
99403013	53.92	0 (0.00%)	4663	4405.9	0.113
99403014	8.37	100 (1.48%)	4470	4340.1	0.058
99403016	21.26	0 (0.00%)	4820	4405.9	0.182
99403017	7.68	86 (1.27%)	4625	4348.0	0.123
99403018	17.11	2 (0.02%)	4415	4404.4	0.005
99404732	49.85	0 (0.00%)	4546	4405.9	0.061
99404736	7.24	148 (2.20%)	4349	4306.5	0.019
99404738	3.81	681 (10.13%)	4244	3958.8	0.139
99404739	14.42	15 (0.22%)	4693	4395.9	0.131
99404740	8.85	10 (0.14%)	4770	4399.4	0.163
99404742	13.26	10 (0.14%)	4711	4400.0	0.137
99404743	19.80	0 (0.00%)	4066	4405.9	-0.149
99404744	28.66	0 (0.00%)	4065	4405.9	-0.150
99404746	27.65	0 (0.00%)	4933	4405.9	0.231
99404749	12.74	13 (0.19%)	4333	4397.6	-0.028
99404750	9.89	54 (0.80%)	4620	4370.6	0.110
99404751	3.02	1061 (15.78%)	4098	3718.7	0.199
99404752	9.07	3 (0.04%)	4161	4403.5	-0.106
99404753	4.61	463 (6.88%)	4792	4103.5	0.325
99404755	8.09	103 (1.53%)	4372	4340.6	0.014
99404756	36.02	0 (0.00%)	4422	4405.9	0.007
99404757	5.73	258 (3.83%)	4616	4234.8	0.174
99404758	8.88	58 (0.86%)	4134	4369.1	-0.104
99404759	4.89	406 (6.03%)	4816	4132.4	0.318
99404761	14.09	9 (0.13%)	4385	4399.4	-0.006
99404762	3.99	283 (4.21%)	4729	4226.3	0.231
99404763	4.11	659 (9.80%)	4385	3975.2	0.200
99404764	19.72	0 (0.00%)	4363	4405.9	-0.019
99404765	7.36	155 (2.30%)	4821	4304.3	0.232
99404799	17.45	1 (0.01%)	4387	4405.4	-0.008
CROW-6	11.96	1 (0.01%)	4167	4404.9	-0.104
CROW-7	10.46	19 (0.28%)	4308	4393.1	-0.037
CROW-8	7.00	106 (1.57%)	4567	4336.3	0.103
CROW-9	14.46	0 (0.00%)	3589	4405.9	-0.359
CROW-21	15.00	1 (0.01%)	4218	4405.2	-0.082
CROW-24	18.73	0 (0.00%)	4463	4405.9	0.025

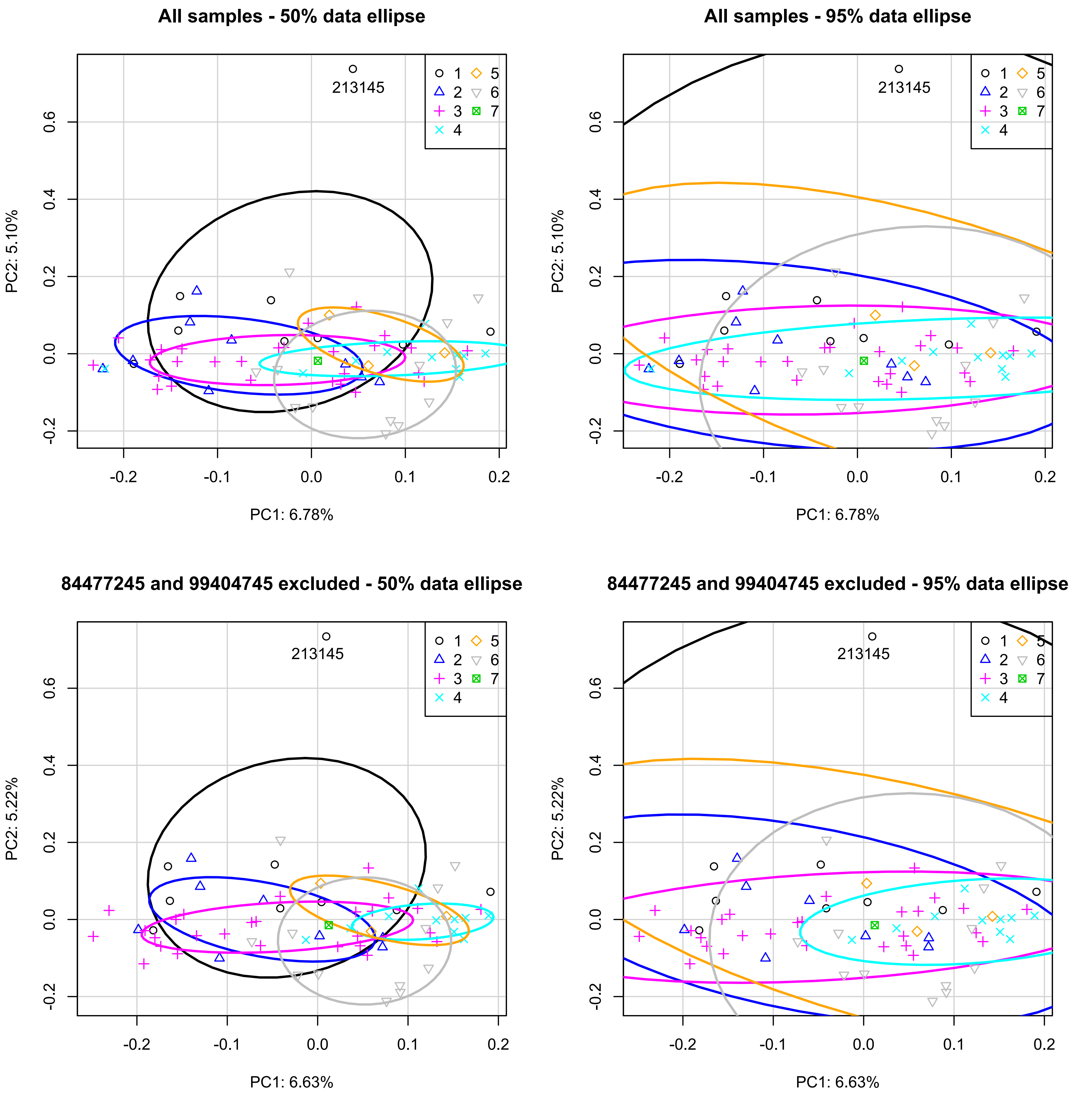

All samples - 50% data ellipse
All samples - 95% data ellipse
84477245 and 99404745 excluded - 50% data ellipse
84477245 and 99404745 excluded - 95% data ellipse
213145
1
2
3
4
5
6
7
PC1: 6.78%
PC2: 5.10%
PC1: 6.63%
PC2: 5.22%
-0.2
-0.1
0.0
0.1
0.2
0.4
0.6

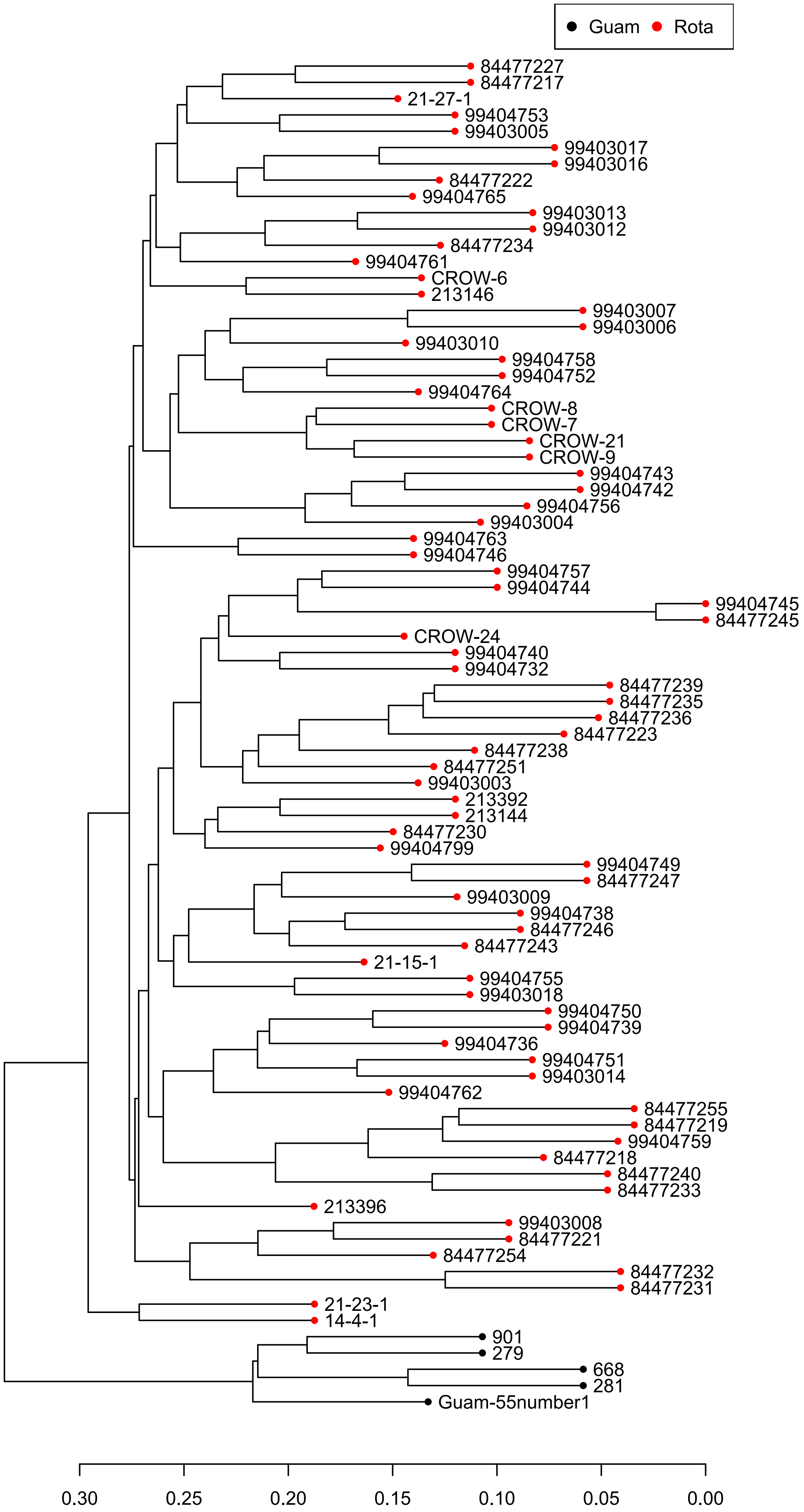

Guam
Rota
84477227
84477217
21-27-1
99404753
99403005
99403017
99403016
84477222
99404765
99403013
99403012
84477234
99404761
CROW-6
213146
99403007
99403006
99403010
99404758
99404752
99404764
CROW-8
CROW-7
CROW-21
CROW-9
99404743
99404742
99404756
99403004
99404763
99404746
99404757
99404744
99404745
84477245
CROW-24
99404740
99404732
84477239
84477235
84477236
84477223
84477238
84477251
99403003
213392
213144
84477230
99404799
99404749
84477247
99403009
99404738
84477246
84477243
21-15-1
99404755
99403018
99404750
99404739
99404736
99404751
99403014
99404762
84477255
84477219
99404759
84477218
84477240
84477233
213396
99403008
84477221
84477254
84477232
84477231
21-23-1
14-4-1
901
279
668
281
Guam-55number1
0.30
0.25
0.20
0.15
0.10
0.05
0.00

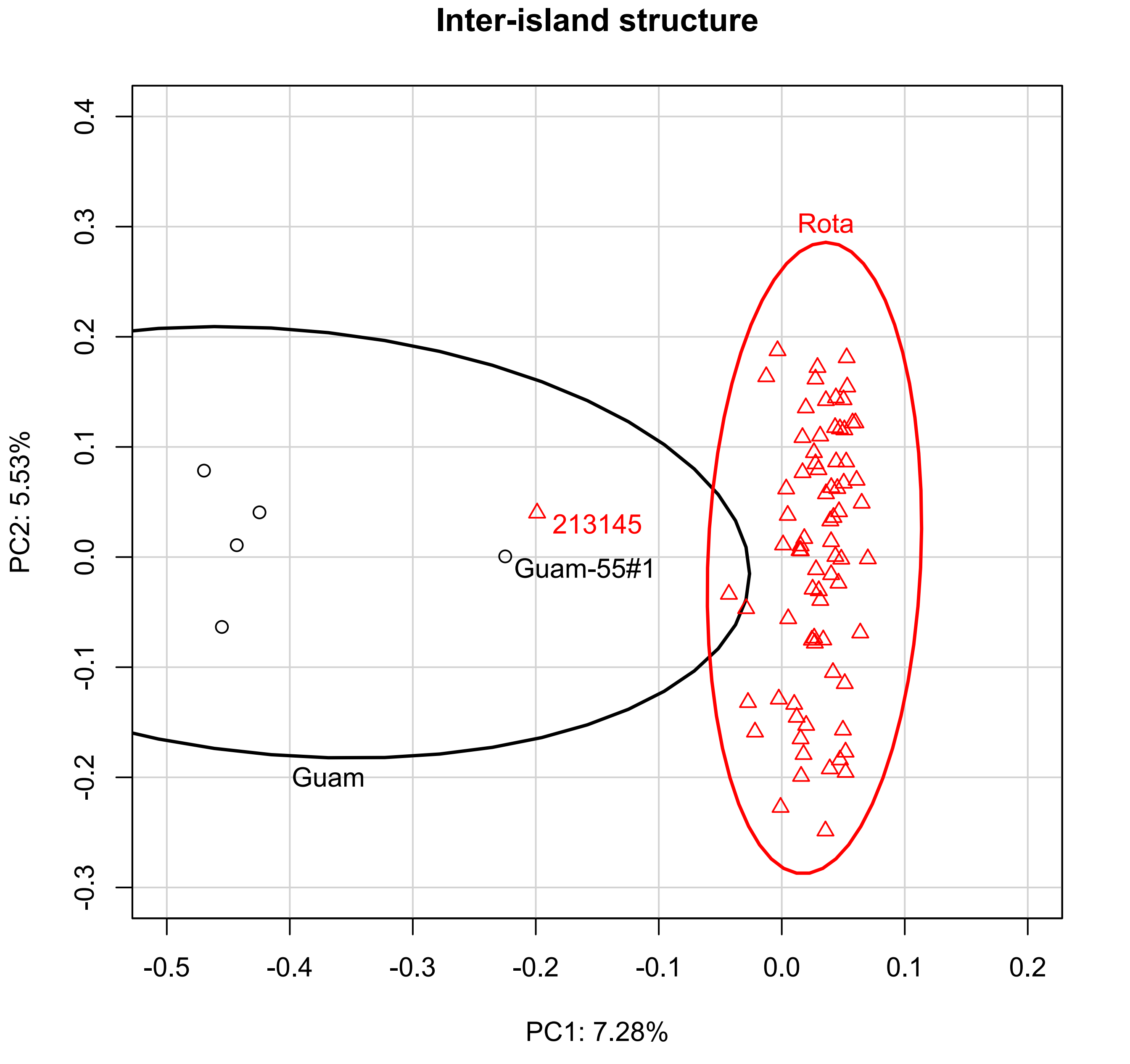
Inter-island structure
Rota
213145
Guam-55#1
Guam
PC1: 7.28%
PC2: 5.53%
-0.5
-0.4
-0.3
-0.2
-0.1
0.0
0.1
0.2
0.4
0.3
0.2
0.1
0.0
-0.1
-0.2
-0.3

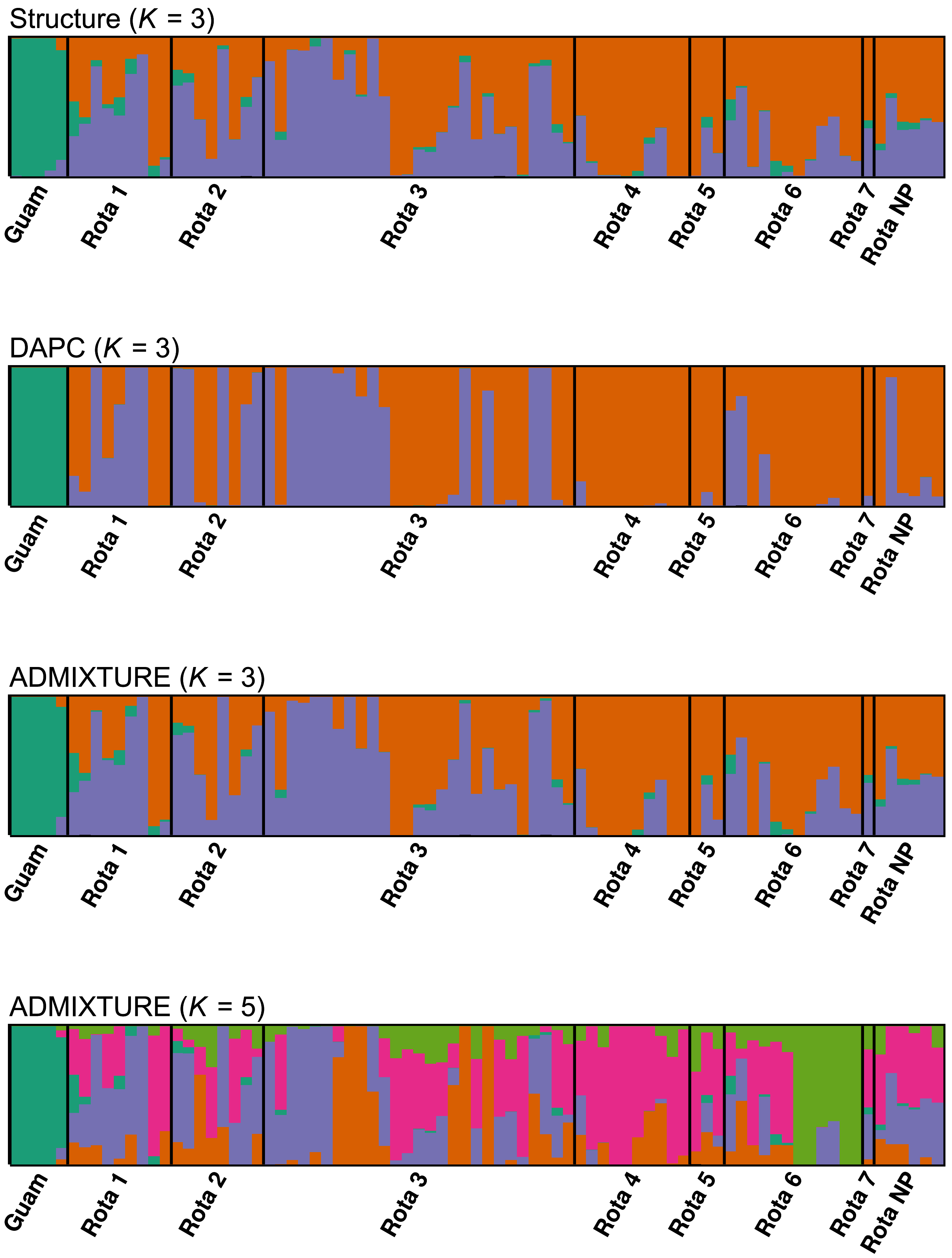
Structure (K = 3)
Guam
Rota 1
Rota 2
Rota 3
Rota 4
Rota 5
Rota 6
Rota 7
Rota NP
DAPC (K = 3)
Guam
Rota 1
Rota 2
Rota 3
Rota 4
Rota 5
Rota 6
Rota 7
Rota NP
ADMIXTURE (K = 3)
Guam
Rota 1
Rota 2
Rota 3
Rota 4
Rota 5
Rota 6
Rota 7
Rota NP
ADMIXTURE (K = 5)
Guam
Rota 1
Rota 2
Rota 3
Rota 4
Rota 5
Rota 6
Rota 7
Rota NP
